# High-starch diets alter equine faecal microbiota and increase behavioural reactivity

**DOI:** 10.1038/s41598-019-54039-8

**Published:** 2019-12-09

**Authors:** Louise S. Bulmer, Jo-Anne Murray, Neil M. Burns, Anna Garber, Francoise Wemelsfelder, Neil R. McEwan, Peter M. Hastie

**Affiliations:** 1 School of Veterinary Medicine, College of Medical, Veterinary and Life Sciences, University of Glasgow, Glasgow, G61 1QH UK; 2 Institute of Biodiversity, Animal Health and Comparative Medicine, University of Glasgow, Glasgow, G12 8QQ UK; 3 Animal and Veterinary Sciences, SRUC, Roslin Institute Building, Midlothian, EH25 9RG UK; 4 School of Pharmacy and Life Sciences, Robert Gordon University, Aberdeen, AB10 7GJ UK

**Keywords:** Microbiome, Animal behaviour

## Abstract

Gut microbiota have been associated with health, disease and behaviour in several species and are an important link in gut-brain axis communication. Diet plays a key role in affecting the composition of gut microbiota. In horses, high-starch diets alter the hindgut microbiota. High-starch diets are also associated with increased behavioural reactivity in horses. These changes in microbiota and behaviour may be associated. This study compares the faecal microbiota and behaviour of 10 naïve ponies. A cross-over design was used with experimental groups fed high-starch (HS) or high-fibre (HF) diets. Results showed that ponies were more reactive and less settled when being fed the HS diet compared to the HF diet. Irrespective of diet, the bacterial profile was dominated by two main phyla, *Firmicutes*, closely followed by *Bacteroidetes*. However, at lower taxonomic levels multivariate analysis of 16S *rRNA* gene sequencing data showed diet affected faecal microbial community structure. The abundance of 85 OTUs differed significantly related to diet. Correlative relationships exist between dietary induced alterations to faecal microbiota and behaviour. Results demonstrate a clear link between diet, faecal microbial community composition and behaviour. Dietary induced alterations to gut microbiota play a role in affecting the behaviour of the host.

## Introduction

Gut microbiota and its effect on behaviour, health and disease has become a major area of scientific interest. The two-way communication system linking the gut and brain is commonly referred to as the gut-brain axis. Growing evidence indicates that gut microbiota play a pivotal role in this communication system^1^. Germ-free mice have been shown to exhibit elevated levels of plasma adrenocorticotropic hormone and corticosterone in response to stress, which was reversed following colonisation of the gut with *Bifidobacterium infantis*^2^. *B*. *infantis* has also been shown to reduce the behavioural and biochemical changes in rats that occurred following a postnatal maternal separation experiment^3^. In humans, patients presenting with both gastrointestinal disorders and stress-related mental health conditions like anxiety and depression have been reported in several studies^1,4–6^. The composition of gut microbiota may play a pivotal role in the connection between these conditions.

Diet is a key factor influencing the composition of gut microbiota^1^. Mice transplanted with gut microbiota associated with a high-fat diet showed increased intestinal barrier permeability along with disruptions in brain physiology and function^7^. The overflow of undigested carbohydrates into the hindgut has also been associated with increased anxiety and depression in rats^8^. In equids, there has been a longstanding anecdotal association between high-starch diets and increased behavioural reactivity. The horse has a digestive tract that is well suited to high-fibre, low-starch diets^9–11^. However, this mostly forage diet is often reduced and substituted with high-starch feeds as a means of increasing energy requirements to fuel athletic performance. These factors make the horse ideal for studying the effects of dietary changes on gut microbiota and behaviour.

A recent behavioural study using mature horses receiving isoenergetic diets found that their heart rates were significantly higher when fed a high-starch diet compared to a high-fibre diet^12^. The hindgut of the horse is abundant in microorganisms^13,14^ and studies have shown that a high-starch diet can alter the equine hindgut microbiota^15–19^. It is likely that alterations to gut microbiota may elicit behavioural changes via gut-brain axis pathways. Increased stress responses have been associated with changes in gut microbiota in fistulated horses^20^. The gastrointestinal tract releases over 20 different hormones^21^, several of which are neurotransmitters^1,22^ and, therefore, any disruption of these neurotransmitters may have a wider influence on the behaviour of the host.

The aims of this study were: to investigate behavioural differences in groups of naïve ponies fed diets high in starch or high in fibre, and to use 16S *rRNA* gene sequencing of faecal samples to determine if there were any alterations to the faecal microbiota associated with the two diets. Finally, we explored the link between diet, gut microbiota and behaviour.

## Results

### Quantitative behavioural analysis

#### Passive human test results

There was a significant increase in the number of times the ponies changed pace when fed the HS diet compared to the HF diet in the passive human test (*z* = 4.22, *p* < 0.01). Additionally, ponies spent less time standing (*t* = −3.63, *p* = 0.01), and less time investigating their surroundings when fed the HS diet (*t* = −2.57, *p* < 0.05). There was no effect of experimental period on the behavioural variables for this test.

#### Novelty test results

Ponies fed the HS diet showed an increase in the number of times they demonstrated a heightened alert state in the novelty test (*z* = 3.49, *p* < 0.01). In contrast to the passive human test there was an effect of experimental period on being alert. Ponies demonstrated increased frequencies of being alert during the second period compared to the first (*z* = −3.32, *p* < 0.01). A significant interaction between diet and experimental period was also retained in this model (*z* = −2.39, *p* < 0.05). The interaction between these terms showed ponies that received the HF diet in period 2 displayed a reduced frequency of alert behaviours but to a lesser extent than those that received the HF diet in period 1.

### Qualitative behavioural analysis

#### Passive human test results

The percentage of variation between observer scoring configurations explained by the GPA consensus profile was 74.1%. There was a significant difference between the GPA consensus profile and the variation explained by the mean randomised profile (*t*_99_ = 36.83, *p* < 0.001). Dimension one explained 56% of the variation. This dimension was defined by strongly correlating, frequently used terms such as relaxed/laid-back/settled at the positive end and nervous/tense/unsure at the negative end (Table 1). This suggests that dimension one characterised how relaxed and settled, or, how nervous and tense the ponies were. There was a significant effect of diet on dimension one (*t* = − 4.22, *p* < 0.01). Ponies were perceived as more tense/nervous/unsure when fed the HS diet.

**Table 1:** Passive human test QBA dimensions, showing observer terms and significance related to diet and period.

GPA dimension (variance explained)	Low value terms r < -0.5	High value terms r > 0.5	Significance related to diet	Significance related to experimental period
1 (56%)	**Nervous** (6), **Tense** (7), **Unsure** (6), Stressed (5), Anxious (5), Alert (4), Excitable (4), Scared (4), Worried (3), Spooked (3), Flighty (3), Jumpy (2), On-Edge (2), Restless (2), Shy (2), Energetic (2), Agitated, Confused, Annoyed, Uptight, Terrified, Upset, Panicked, Frightened, Agitated, Skittish, Cautious, Dominant, Sharp, Skatty, Afraid, Aware	**Relaxed** (10), **Laid-back** (8), **Settled** (7), Calm (6), Happy (6), Easy-going (2), Content (2), Chilled (2), Gentle, Comfortable, Unphased, Bored, Quiet, Carefree	*p* < 0.01	n.s.
2 (15.5%)	**Wary** (4), **Scared** (2), **Hesitant** (2), Nervous (2), Unsure (2), Shy (2), Anxious, Cautious, Apprehensive, Interactive	**Bold** (8), **Confident** (6), **Brave** (4), Curious (2), Playful (3), Inquisitive, Nosey, Excited, Interested, Determined	n.s.	n.s.

Included are observer terms correlating strongly with those dimensions at the negative end (r < −0.5) and positive end (r > 0.5). Values in brackets following terms indicate the number of observers using that term if greater than 1. The terms in bold were used to label the dimensions.

Dimension two explained 15.5% of the variation and ranged from bold/confident/brave to wary/scared/hesitant, suggesting that this dimension characterised how ‘confident’ ponies were in responding to the test. There was no effect of diet on dimension two and no effect of period on either dimension for this test.

Spearman’s rank correlation coefficient showed a moderate and positive correlation between dimension one and the quantitative variable time spent investigating (n = 20, *ρ* = 0.56, *p < *0.05). This indicates that ponies that explored more were perceived as relaxed/laid-back/settled. There was also a moderate negative correlation between dimension one and the quantitative variable frequency of pace-change (n = 20, *ρ* = −0.51, *p < *0.05) indicating that ponies that changed pace more frequently were perceived as nervous/tense/unsure.

#### Novelty test results

The percentage of variation between observer scoring configurations explained by the GPA consensus profile was 70.2%. The GPA consensus profile differed significantly from the mean randomised profile (*t*_99_ = 31.33, *p* < 0.001). Dimension one explained 54.1% of the variation and characterised behaviours ranging from relaxed/laid-back/settled to nervous/unsure/tense (Table 2). Dimension two explained 13.3% of the variation and characterised behaviours like bold/curious/confident at the positive end. “Shy” was the only term at the negative end. There was no significant effect of diet on either dimension for the novelty test. However, there was an effect of experimental period on dimension one. Ponies were perceived as more nervous/unsure/tense in response to the foil-covered box used in period one, than in response to the triangular road sign used in period two.

**Table 2:** Novelty test QBA dimensions, showing observer terms and significance related to diet and period.

GPA dimension (variance explained)	Low value terms r < −0.5	High value terms r > 0.5	Significance related to diet	Significance related to experimental period
1 (54.1%)	**Nervous** (8), **Unsure** (7), **Tense** (6), Scared (6), Anxious (6), Restless (5), Wary (5), Spooked (4), Alert (4), Stressed (4), Worried (3), On-edge (3), Hesitant (2), Agitated (2), Confused (2), Annoyed, Shy (2), Cautious (2), Uptight, Terrified, Upset, Panicked, Frightened, Excited, Skittish, Jumpy, Careful, Flighty, Sharp, Skatty, Afraid, Uncertain	**Relaxed** (10), **Laid-back** (8), **Settled** (7), Happy (7), Calm (6), Bold (3), Confident (2), Brave (2), Content (2), Chilled (2), Easy-going, Bored, Carefree, Comfortable, Interactive, Pre-occupied, Nosey, Unphased	n.s.	*p* < 0.01
2 (13.3%)	Shy	**Bold** (7), **Curious** (5), **Confident** (5), Jumpy (3), Brave (4), Inquisitive (4), Flighty (2), Spooky (2), Alert, Energetic, Courageous, Determined	n.s.	n.s.

Included are observer terms correlating strongly with those dimensions at the negative end (r < −0.5) and positive end (r > 0.5). Values in brackets following terms indicate the number of observers using that term if greater than 1. The terms in bold were used to label the dimensions.

### 16S *rRNA* gene sequencing

**Figure 1:**
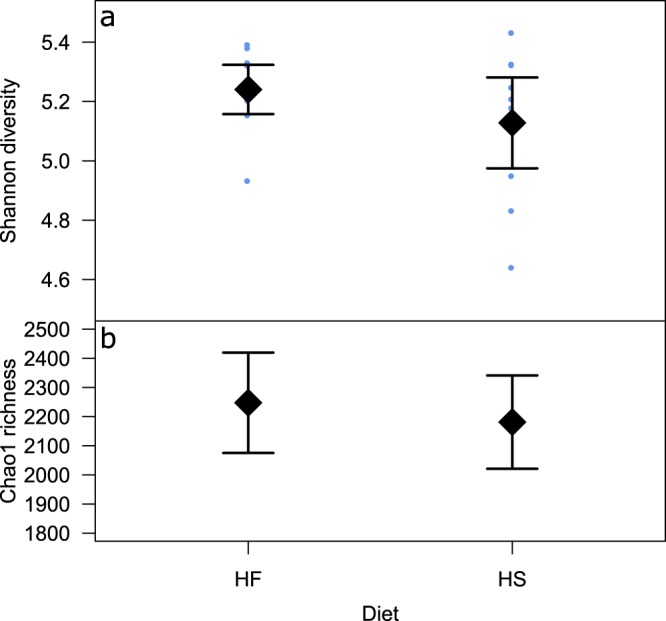
Shannon and Chao indices. Comparison of diversity and richness indices for high-starch (HS) and high-fibre (HF) diets. Error bars show 95% confidence interval. Points on plot (**a**) denote individual Shannon diversity measures for each pony with the measures for the HS diet showing greater variance.

Results from the 20 faecal samples sequenced gave a total count of 1,251,244 reads, with an average of 65,026 ± 12,095 (mean ± s.d.) reads per sample for the HF diet and 60,098 ± 17,780 (mean ± s.d.) reads per sample for the HS diet. Shannon diversity results showed similar values for the HF and HS diets (Fig. 1), but overall diversity was lower and showed greater variance for the HS diet compared to the HF diet. The Chao1 richness estimator also showed similar values for both diets but overall richness was lower for the HS diet compared to the HF diet (Fig. 1).

### Microbial community composition

**Figure 2:**
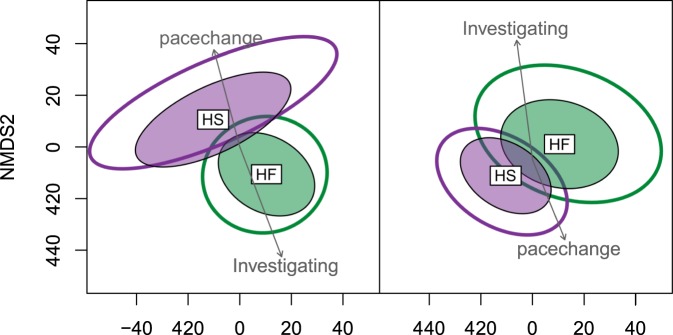
NMDS plots comparing community structure for the HS (high-starch) and HF (high-fibre) diets for testing period 1 (**a**) and testing period 2. (**b**) The significant behavioural results from the passive human test (time spent investigating and frequency of pace change) have been plotted over the top of the diets showing the direction towards which these behaviours are seen to increase. The arrows point towards the direction of increasing gradient with the length of the arrows indicating the proportion of correlation. The frequency of pace-change increases in the direction of the HS diet while the time spent investigating increases in the direction of the HF diet.

NMDS ordination plots showed grouping of microbial community structure related to diet for both experimental periods (Fig. 2). While some overlap was evident in the ordination plots, the community structure also showed differences related to diet. This was consistent for both testing periods; however, period one did show wider variation in community structure for the HS diet compared to the HF diet.

A clear relationship was visible between the microbial community structure and the behaviour of the ponies when plotted on the ordinations plots in both experimental periods. Time spent investigating increased in the direction of the HF diet and the frequency of pace-change increased in the direction of the HS diet (Fig. 2). Results showed strong correlations for each of the behavioural variables with faecal microbial community composition.

**Figure 3:**
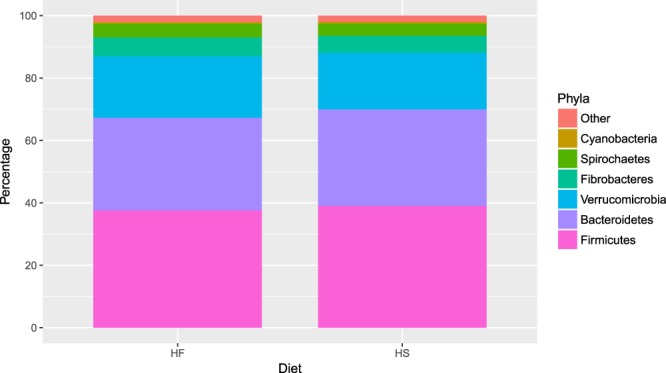
Bacterial taxa plot showing phyla level relative abundance for the high-starch (HS) and high-fibre (HF) diets.

16S *rRNA* gene sequencing data identified a total of five main phyla, which in terms of abundance, represented >97% of the overall microbial profile from the faecal samples (Fig. 3). Each of the five phyla represented >1% of the total population. Irrespective of diet, the overall profile was dominated by two main phyla, *Firmicutes*, closely followed by *Bacteriodetes*.

For the faecal microbial community composition analysis, model selection resulted in an optimal multivariate model which included the explanatory variables, ‘diet’, experimental period’ and the interaction between diet and period. This model was significantly better than the simpler nested model which did not include the interaction (*p < *0.01, Wald = 66.68). Cross validation, comparing the optimal model predictions of OTU abundance with true OTU abundance showed a strong and significant correlation (*ρ* = 0.75, *p < *0.001) between the prediction and true values.

**Figure 4:**
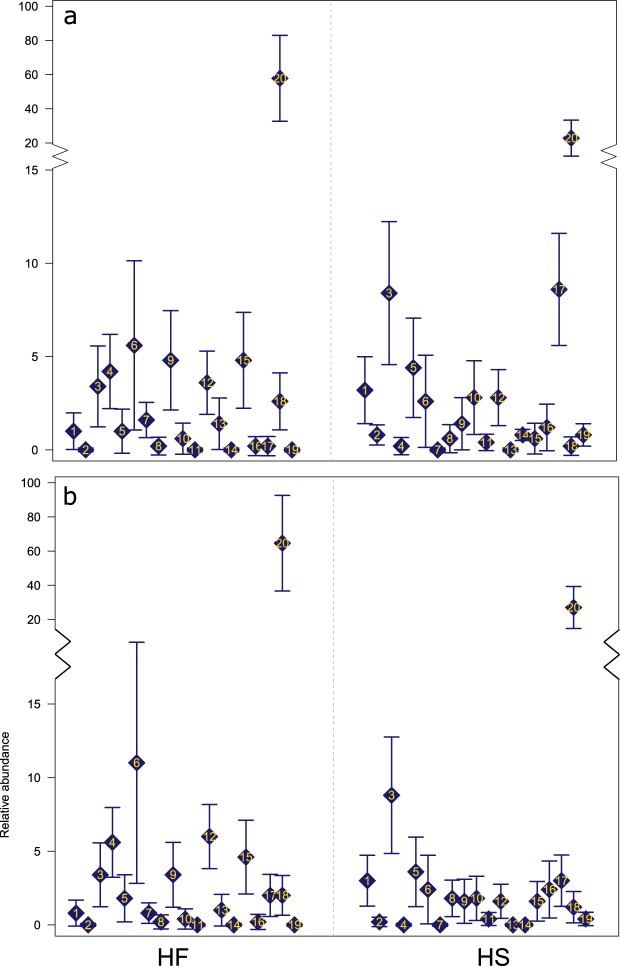
OTU abundance plots for both experimental periods illustrating the relative abundance of individual OTUs related to diet (n = 20, α = 99%). Panel (a) shows experimental period 1 and panel (b) shows experimental period 2. OTU identification: 1 = *Firmicutes-Lachnospiraceae*, 2 = *Proteobacteria-Alphaproteobacteria*, 3 = *Firmicutes-Lachnospiraceae*, 4 = *Firmicutes-Oscillospira*, 5 = *Bacteroidetes-Bacteroidales*, 6 = *Firmicutes-Ruminococcaceae*, 7 = *Firmicutes-Ruminococcaceae*, 8 = *Firmicutes-Clostridiales*, 9–12 = *Firmicutes-Ruminooccaceae*, 13 = *Firmicutes–Lachnospiraceae*, 14 & 15 = *Firmicutes-Clostridiales*, 16 = *Firmicutes-Streptococcus*, 17 = *Firmicutes-Oscillospira*, 18 = *Firmicutes-Christensenallaceae*, 19 = *Firmicutes-Oscillospira*, 20 = *Firmicutes-Lachnospiraceae*.

Multivariate GLMs showed that the abundance of several OTUs was significantly affected by diet (Table 3). Results also showed an effect of the interaction between diet and period on the abundance of OTUs. Diet had a significant effect on the abundance of 85 OTUs regardless of experimental period (*p* < 0.05). Figure 4 displays the 20 OTUs which show a significant difference depending on diet at *p* < 0.001. Of these 20 OTUs 18 were from the *Firmicutes* phylum, one was from the *Bacteroidetes* phylum and one from the *Proteobacteria* phylum. *Proteobacteria* represented less than 1% of the overall relative abundance. From the OTUs of the *Firmicutes* phylum, 17 were from the *Clostridia* class and *Clostridiales* order, with the remaining OTU coming from the *Bacilli* class and *Lactobacilli* order.

**Table 3:** Model output from 16 S *rRNA* gene sequencing multivariate model (mvabund).

Test statistics	Wald value	P-value
(Intercept)	191.36	<0.001
DietHS	40.38	0.015
Period2	32.64	0.261
DietHS:Period2	39.18	0.013

Results show a significant effect of diet on community composition and a significant interaction between diet and experimental period on community composition.

From the 17 OTUs of the *Clostridiales* order, three were from the *Lachnospiraceae* family and were unassigned at genus level, and ten were from the *Ruminococcaceae* family with three of these assigned to the *Oscillospira* genus with the remainder unassigned at genus level. Of the four remaining OTUs from the *Clostridiales* order, three were unassigned at family level and one was from the *Christensenellaceae* family. *Christensenallaceae was significantly decreased with the HS diet compared to the HF diet regardless of experimental period*. The remaining OTU from the *Firmicutes* phylum (*Bacilli* class and *Lactobacillales* order) was identified to genus level as *Streptococcus* and showed greater abundance in the HS diet than the HF diet regardless of experimental period. The OTU from the *Bacteroidetes* phylum was from the *Bacteroidia* class and *Bacteroidales* order but was unassigned below this level. The OTU from the *Proteobacteria* phylum was from the *Alphaproteobacteria* class and was unassigned below this level.

## Discussion

Results indicate a clear effect of diet on the faecal microbiota and behaviour of naïve ponies. They also showed a strong association between behaviour and faecal microbial profile related to diet. An increase in the frequency of pace-change was closely associated with a HS diet. An increase in time spent investigating was closely associated with a HF diet. The faecal microbial profile from the HS diet is associated with increased behavioural reactivity, while the faecal microbial profile from the HF diet is associated with more settled behaviours. The increase in reactive behaviours seen here with additional starch in the diet may make horses less predictable in their behaviour and therefore more difficult to handle.

When fed the HS diet ponies were more alert and reactive. Conversely when they were fed the HF diet the ponies were more settled. The increased frequency of pace-change with the HS diet shows that ponies were more unsettled and reactive. The QBA assessment also supports this, with observers perceiving the ponies to be more nervous/tense/unsure on the HS diet. It has been reported previously that horses with lower stress levels display less time in locomotion^23^. Conversely, increased “flightiness” has been associated with horses that were most fearful^24^. The young ponies used in the current study may have found the presence of a person in the testing enclosure potentially threatening. Increased locomotory behaviour has been seen previously in young horses fed a starch diet compared to youngsters which received an alternative fat and fibre diet^25^. Increased behavioural reactivity may generally be more obvious when younger horses are studied, with mature horses, over time, learning to become less reactive. This does not mean that mature horses will not react but that it just may not be so obviously displayed and could therefore be more difficult to quantify in a behavioural testing situation.

The results also showed that ponies spent significantly less time investigating their surroundings when fed the HS diet compared to the HF diet. This, like the increase in pace-change results, suggests that ponies were less at ease and unsettled. The QBA assessment also supports this, with observers perceiving the ponies to be more nervous/tense/unsure. The correlation between the quantitative and qualitative measures indicates an overall picture of increased reactivity and unease when ponies were receiving the HS diet compared to the HF diet; as the time spent investigating decreased the QBA assessment of the ponies was perceived as being more nervous/tense/unsure. Changes in investigative behaviours have been observed previously in relation to diet. One study found that youngsters receiving a fat and fibre diet appeared to be less distressed and were more attentive to their environment than youngsters receiving a starch-sugar diet^25^. A reduction in investigative behaviours was also seen in mature horses; when they received a high grain diet they spent less time exploring their stabled environment^26^. However, results from the current study not only show that changes in behaviour related to diet could be measured quantitatively, they also show that the behavioural differences were displayed clearly enough for observers to see.

In the novelty test, ponies showed an increased frequency of being alert when they were fed the HS diet compared to the HF diet. Again, this suggests that the ponies were less at ease. In the current study, some food in a bucket was in place during the novelty test to motivate ponies to approach the novel object. This motivation to approach a novel object is important so that the animal’s response to it can be interpreted^27^. There was no significant effect of diet on the frequency with which the ponies glanced at the feed bowl suggesting that there was no difference in their motivation to eat. Our result contrasts with an earlier study that found weanlings being fed a fat and fibre based diet spent significantly more time investigating a novel object compared to those fed a starch-sugar diet^25^. As a flight animal, a horse’s response to an unfamiliar object cannot always be anticipated and the difference in results may be due to differences in the novel objects used. QBA analysis found that ponies were more nervous/tense/unsure in experimental period 1. This could be due to the different novel objects used for the two experimental periods. It is possible that the different objects were not equally alarming to the ponies. There was no effect of diet, however, on the ponies’ responses to either object.

Multivariate analyses results showed a significant difference in microbial community composition related to diet even though richness and diversity measures showed similarity. Therefore, it is the composition of the bacterial taxa that contribute to the overall diversity which differed in relation to diet. The OTUs that were identified as showing significant differences in abundance related to diet mostly originated from the *Firmicutes-Clostridia-Clostridiales* lineage, with the majority coming from the *Ruminococcaceae* family. *Ruminococcaceae* bacteria have previously been identified as fibrolytic bacteria^28^. Our study shows that even a small addition of starch to the diet is enough to have an effect on reducing this bacterial population. Research has shown that the extent of the alterations to faecal microbiota with the addition of starch to the diet can be influenced by the source of starch fed^29^.

An increase in lactic-acid producing bacteria has also been reported to be coupled with a corresponding decrease in fibrolytic bacterial abundance^28^. Another of the OTUs that was significant different related to diet was *Streptococcus*, which showed a higher relative abundance for the HS diet compared to the HF diet. *Streptococcus*, from the *Firmicutes-Bacilli-Lactobacillales* taxonomic lineage, is a lactic-acid producing bacteria^16^ and has been reported to be the predominant bacteria seen to proliferate prior to the onset of oligofructose-induced laminitis^30^. In previous studies, *Streptococcus* and *Lactobacilli*, have been reported to progressively increase in response to dietary change^28,31^, with lower proliferation when there was less starch in the diet^16^. In the current study, starch was fed at a level of 1 g/kg BW/meal for the HS diet and the faecal microbiota still showed an increase in *Streptococcus* bacteria. This shows that even small additions of starch to the diet, which should not pass through the small intestine undigested, can still result in an increase of *Streptococcus* bacteria. However, it is less clear whether an increase in *Streptococcus* at these levels has any wider health implications for the host, or if this is an adaptation brought about by the addition of starch to a mostly fibre-based diet.

A study using mice found that dietary induced alterations to microbiota resulted in significant differences in behaviour^32^. In horses, a recent study using culture-based techniques found correlations between alterations in gut microbiota and an increased stress response^20^. However, a greater quantity of starch was fed meaning that it may have passed undigested into the hindgut. It is therefore possible that the increased stress response might have been associated with digestive discomfort. In the current study it is unlikely that starch would enter the hindgut undigested as the feeding levels were within recommended daily maximum levels. Therefore, what remains to be determined are the mechanisms behind the associated behavioural and microbiota changes that were observed in the current study.

What is clear from the current study is that dietary change resulted in alterations in behaviour and faecal microbiota. These changes occurred by making a small dietary adjustment. It is likely that alterations to diet may also initiate similar effects in other species. The increased starch in the current study had an undesirable effect on behaviour and gut microbiota; it made the ponies more reactive in their behaviour and moved the microbial community composition of the gut towards dysbiosis. However, the opposite was true of the HF diet. This diet resulted in ponies displaying more settled behaviour and gut microbiota more in keeping with a healthy equine gut. Therefore, diet may also have a positive, beneficial effect to the host. As such the wider effects of diet on the host should not be underestimated. What is unclear from this study is where in the hindgut the differences observed in the faecal samples originated and, if there are particular regions of the hindgut that are affected by dietary change more than others.

The gut-brain axis encompasses a number of different communication systems, including direct communication via neural pathways and also indirect communication via endocrine and inflammatory pathways^1^. Behavioural and neurochemical changes have been observed in rats following antibiotic induced modulation of gut microbiota^33^. It is possible that the changes in behaviour found in the current study were triggered by neuroendocrine alterations brought about by dietary induced changes to gut microbiota.

## Methods

### Study design

The experimental protocols in this study were approved by the University of Glasgow’s School of Veterinary Medicine Veterinary Ethics & Welfare Committee (Equine hindgut health, nutrition and microbiota – Ref. 05a/14). Consent was obtained from all behavioural observers, and from the passive human behavioural test assistants. All methods were carried out in accordance with the obtained approval and the University of Glasgow’s experimental protocol guidelines.

Ten unhandled 18-month-old Welsh section A ponies were used in a 2 × 2 cross-over design consisting of two 14-day periods and two experimental diets; high-fibre (HF) or high-starch (HS). The ponies were divided into two groups each comprising two fillies and three geldings. In experimental period one, five ponies received the HS diet and five received the HF diet. At the end of the first experimental period the ponies were transitioned to the alternative diet by graduating four feeds over two days. Ponies were housed in individual pens in an indoor barn and bedded on wood shavings. Prior to the study the ponies were fed hay with no additional feed supplementation.

All ponies were fed according to National Research Council^34^ recommendations for growth using the estimated mature bodyweight (BW) of 250 kg (Table 4). The HF diet consisted of hay plus high-temperature dried Lucerne with ponies receiving 0.46 g starch/kg of BW per meal. The HS diet consisted of hay and a compound mix with ponies receiving 0.96 g starch/kg of BW per meal. Both diets were fed in two daily meals. All ponies received 40.2 MJ/DE (Mega joules of digestible energy) per day.

**Table 4:** Energy and starch composition of feeds.

Feed	MJ/DE per kg	Starch content %
Hay	7.5	3.6
Compound mix	10.0	23.0
Lucerne	10.0	5.0

### Behavioural tests

Two behavioural tests were conducted at the end of each experimental period in a testing area (length 10.12 m × width 6.92 m × height 3 m) constructed from straw bales in an indoor barn. The ponies were familiarised with the test area prior to the study. A video camera was positioned on top of the bales at the far end of the testing area. Both tests were recorded with each lasting for five minutes.

A sample of freshly voided faeces was collected for each pony on both behavioural testing days (n = 20) between 10:00 am and 12:00 noon. Care was taken to collect samples from the centre of the faeces that had not been in contact with the floor or bedding material. Samples were immediately frozen at −20 °C and then stored at −80 °C prior to genomic DNA extraction (gDNA).

### Passive human test

The passive human test^35^ was carried out using a person unfamiliar to the ponies stood centrally 1 m in from the right hand wall (when viewed from camera) of the testing area. A different person was used for each behavioural testing period to ensure that the ponies did not become familiar with the person. A pony was released into the testing area and their behavioural responses to the motionless human were recorded. This test was conducted following the handling protocols approved by the University of Glasgow (Equine hindgut health, nutrition and microbiota – Ref. 05a/14).

### Novelty test

For the novelty test^36^, a pony was released into the testing area which contained a black rubber feed bowl and a novel object. The feed bowl contained a mixture of Lucerne and compound mix and was positioned centrally 1.6 m in from the end wall. A novel object (1st period = large box (86 cm × 44 cm × 20 cm) wrapped in foil, and 2nd period = triangular road sign) was placed between the feed bowl and the entrance at 1.6 m from the feed bowl.

### Quantitative behaviour measures

Data from the behavioural variables (Table 5) were extracted from the video recordings using Observer XT (version 12.5) Software (Noldus Information Technology). The locomotory variables were categorised as continuous and mutually exclusive. The other behavioural categories were categorised as mutually exclusive.

**Table 5:** Behavioural variables measured during the passive human and novelty tests.

Behaviour	Passive human test	Novelty test
Alertness:		
Glances at human^a,b^(f)	**x**	
Glances at stimulus^b,c^(f)		**x**
Glances at feed bowl^b,^ (f)		**x**
Alert other ^c^(f,d) (as with glancing but ears orientated in other directions)	**x**	**x**
Interaction:		
Investigate human^a,b^(f,d)	**x**	**x**
Investigate stimulus^a,b^(f,d)		**x**
Investigate other^c^(f,d) (includes sniffing, touching and manipulating walls, gate or floor)	**x**	**x**
Feeding:		
Time to approach feed bowl ^e^ (l)		**x**
Sniff food^c^(l,d)		**x**
Time eating^c^ (d) (head may be lifted away from feed bowl for short periods while chewing)		**x**
Locomotion:		
Stand (d)	**x**	**x**
Walking ^c^ (d)	**x**	**x**
Trotting ^a,b,c^ (d)	**x**	**x**
Cantering ^a,b,c^ (d)	**x**	**x**
Pace change (f)	**x**	**x**

Key: ‘x’ indicates for which test each behavioural variable was measured. Types of measure; f = frequency, d = duration, l = latency. (References: a = 35, b = 36, c = 27, e = 12.

### Qualitative behavioural analysis

Qualitative behavioural analysis (QBA) was used to assess the animals’ expressive demeanour as interpreted by a group of observers. Ten undergraduate equine students consented to participate as observers. The observers were unfamiliar with the ponies and unaware of the aim of the study but had at least three years’ experience working with horses. Observer descriptions include terms like bold, curious or nervous^37–42^.

Two minute sections of the behavioural video recordings were used. For the passive human test, minutes 1.5–3.5 were used and for the novelty test the first two minutes were used; ensuring that the element of surprise at the novel object was captured. Free-choice profiling methodology (FCP) was used in two phases. In the first phase the observers each generated their own descriptive vocabularies. The second phase involved the quantification by each observer of their own descriptive terms. The observers watched the video clips in a random order. Following each clip, the observers used their list of descriptive terms to score the observed pony using an unstructured visual analogue scale measuring 125 mm. The extreme left (0 mm) was marked as ‘minimum’ and the extreme right (125 mm) as ‘maximum’. Observers were instructed to use the distance between these points to mark the intensity of expression for each behavioural term.

### DNA extraction and 16S *rRNA* gene sequencing

Genomic DNA (gDNA) was extracted from faecal samples using a Qiagen QIAamp^®^ Fast DNA Stool Mini Kit. The standard protocol was followed but with slight adaptations. These included the samples being immediately homogenised in the InhibitEx^®^ Buffer using a homogeniser. The samples were then incubated at 70 °C for five minutes prior to centrifugation at full speed in a micro-centrifuge (14,000 × *g*) and pipetting into tubes containing Proteinase K^®^. Finally, ATE^®^ Buffer was added directly onto the QIAamp^®^ membrane and incubated at room temperature for five minutes instead of one minute prior to centrifuging.

Extracted gDNA was assayed for concentration, quality and protein contamination using a nanodrop to measure A_260_ and A_280_ values prior to freezing at −20 °C. Samples were briefly thawed and aliquoted according to gDNA concentration, then frozen at −20 °C prior to 16 S *rRNA* sequencing.

Library preparation and sequencing were undertaken by Glasgow Polyomics, University of Glasgow, using the Illumina protocol. Sequencing was run on an Illumina MiSeq using 2 × 300 bp paired end reads. The library preparation used was based upon Illumina’s 16 S library preparation. In brief, primers (forward 5′-CTTACGGGNGGCWGCAG-3′ and reverse 5′-GACTACHVGGGTATCTAATCC-3′) were used to amplify the V3 and V4 regions of the 16S *rRNA* gene, with the addition of Nextera XT V2 indices and adapters during the second round of PCR. These libraries were quality controlled and the equimolar pooled and sequenced. 3.5% PhiX spike-in was added to the run as a sequencing control. Bioinformatic analysis of raw sequencing data were processed by Glasgow Polyomics using QIIME (Quantitative Insights Into Microbial Ecology)^43^. Reads were clustered into operational taxonomic units (OTUs) based upon 97% identity using the default alignment method ‘uclust’. All samples were rarefied to 9000 reads/sample.

### Statistical analyses

#### Quantitative behavioural analysis

Data analyses were carried out using R (3.4.3)^44^. A generalised linear mixed effects model (GLMM) with random intercept was selected using AIC to confirm the optimal random effects structure. Backward stepwise model selection was used to select the optimal fixed effects^45^. Differences in behavioural response were modelled as a function of diet and experimental period using the ‘*nlme*’ package^46^ for Gaussian data (e.g. time spent investigating) and ‘*lme4*’^47^ for Poisson distributed data (e.g. frequency of pace change).

#### Qualitative behavioural analysis

The score for each pony from an observer’s behavioural terms was measured in millimetres. The level of agreement between the observer data matrices was analysed for each test using Generalised Procrustes Analysis (GPA), run by a specialist GenStat software programme developed for F. Wemelsfelder. GPA does not require pre-defined variables; a series of randomised iterative rotations finds a consensus profile or ‘best fit’ for the different observer data configurations^37,40^. A one-tailed t-test was used to determine whether the percentage of variation explained by the ‘true’ consensus differed significantly from the mean of 100 randomised profiles, with a p-value < 0.001 indicating a ‘true’ consensus. Principal Component Analysis (PCA) was then used to identify the main dimensions of the consensus profile explaining the majority of variation between observed animals. These dimensions were interpreted by correlating the individual scoring patterns to the main dimensions of the consensus profile and then collating terms for all observers that correlated with these dimensions (r < −0.5 and r > 0.5).

Analysis of the effect of diet and experimental period on GPA consensus scores for each behavioural test was carried out in R using a GLMM with pony as the random intercept. Spearman’s rank correlation coefficient was then used to determine if any significant correlations were present between quantitative and qualitative behavioural outcomes related to diet.

#### 16S *rRNA* gene sequencing data analyses

Statistical analyses on the microbial dataset were performed in R. Diversity and richness for both diets were evaluated using Shannon diversity (vegan package^48^) and Chao1 richness indices (fossil package^49^). Rarefied data were visualised according to diet and experimental period using NMDS (non-metric multidimensional scaling) plots. To investigate any associations between microbial community structure and behaviour, key behavioural variables were fitted over the microbiota NMDS ordination plots for both experimental periods using the ‘*envfit*’ function in the vegan package^48^.

The multivariate modelling of 16S *rRNA* gene data was undertaken using the mvabund package^50^. This package is a powerful tool with greater power properties than distance-based methods^51^. The ‘*manyglm*’ function with a negative-binomial distribution with log-link was used allowing a generalised linear model (GLM) to be fitted to each OTU^52^. To account for possible correlation between variables, ridge regularisation was applied to shrink the correlation matrix. A full model was fitted with ‘diet’, ‘experimental period’ and the associated interaction. Wald test statistics were used in backwards stepwise model selection. A robust approach was adopted to verify model accuracy using Monte Carlo cross-validation^53^. 10,000 iterations of an 80:20 (training:test) data split were used to compare test set predictions with the true results using a Spearman’s rank correlation coefficient.

### Ethical approval

The experimental protocols in this study were approved by the University of Glasgow’s School of Veterinary Medicine Veterinary Ethics & Welfare Committee (Equine hindgut health, nutrition and microbiota – Ref. 05a/14). Consent was obtained from all behavioural observers, and from the passive human behavioural test assistants. All methods were carried out in accordance with the obtained approval and the University of Glasgow’s experimental protocol guidelines.

## Data Availability

The 16S rRNA gene sequencing dataset for this study is be available online from the University of Glasgow Enlighten database 10.5525/gla.researchdata.804.

## References

[1] Cryan JF, Dinan TG (2012). Mind-altering microorganisms: the impact of the gut microbiota on brain and behaviour. Nature Reviews: Neuroscience.

[2] Sudo N (2004). Postnatal microbial colonization programs the hypothalamic-pituitary-adrenal system for stress response in mice. Journal of Physiology.

[3] Desbonnet L (2010). Effects of the probiotic Bifidobacterium infantis in the maternal separation model of depression. Neuroscience.

[4] Crumeyrolle-Arias M (2014). Absence of the gut microbiota enhances anxiety-like behavior and neuroendocrine response to acute stress in rats. Psychoneuroendocrinology.

[5] Foster JA, McVey Neufeld KA (2013). Gut-brain axis: how the microbiome influences anxiety and depression. Trends in neurosciences.

[6] Holzer P, Reichmann F, Farzi A, Neuropeptide Y, peptide YY (2012). and pancreatic polypeptide in the gut-brain axis. Neuropeptides.

[7] Bruce-Keller AJ (2015). Obese-type Gut Microbiota Induce Neurobehavioral Changes in the Absence of Obesity. Biological psychiatry.

[8] Hanstock TL, Clayton EH, Li KM, Mallet PE (2004). Anxiety and aggression associated with the fermentation of carbohydrates in the hindgut of rats. Physiology & behavior.

[9] Henderson AJ (2007). Don’t fence me in: managing psychological well being for elite performance horses. Journal of Applied Animal Welfare Science.

[10] Hill J (2007). Impacts of nutritional technology on feeds offered to horses: A review of effects of processing on voluntary intake, digesta characteristics and feed utilisation. Animal Feed Science and Technology.

[11] Willing B (2009). Changes in faecal bacteria associated with concentrate and forage-only diets fed to horses in training. Equine Veterinary Journal.

[12] Bulmer L, McBride S, Williams K, Murray J-A (2015). The effects of a high-starch or high-fibre diet on equine reactivity and handling behaviour. Applied Animal Behaviour Science.

[13] Daly K, Stewart CS, Flint HJ, Shirazi-Beechey SP (2001). Bacterial diversity within the equine large intestine as revealed by molecular analysis of cloned 16S rRNA genes. FEMS microbiology ecology.

[14] Biddle AS, Black SJ, Blanchard JL (2013). An *in vitro* model of the horse gut microbiome enables identification of lactate-utilizing bacteria that differentially respond to starch induction. PloS one.

[15] Medina B, Girard ID, Jacotot E, Julliand V (2002). Effect of a preparation of *Saccharomyces cerevisiae* on microbial profiles and fermentation patterns in the large intestine of horses fed a high fiber or a high starch diet. Journal of animal science.

[16] de Fombelle A (2003). Characterization of the microbial and biochemical profile of the different segments of the digestive tract in horses given two distinct diets. British Journal of Animal Science.

[17] Al Jassim RA, Scott PT, Trebbin AL, Trott D, Pollitt CC (2005). The genetic diversity of lactic acid producing bacteria in the equine gastrointestinal tract. FEMS Microbiological Letters.

[18] Julliand V, De Fombelle A, Varloud M (2006). Starch digestion in horses: The impact of feed processing. Livestock Science.

[19] Dougal K (2014). Characterisation of the faecal bacterial community in adult and elderly horses fed a high fibre, high oil or high starch diet using 454 pyrosequencing. PloS one.

[20] Destrez A, Grimm P, Cezilly F, Julliand V (2015). Changes of the hindgut microbiota due to high-starch diet can be associated with behavioral stress response in horses. Physiology & behavior.

[21] Murphy KG, Bloom SR (2006). Gut hormones and the regulation of energy homeostasis. Nature.

[22] Clarke G (2014). Minireview: Gut microbiota: the neglected endocrine organ. Molecular endocrinology.

[23] Benhajali H, Richard-Yris MA, Ezzaouia M, Charfi F, Hausberger M (2009). Foraging opportunity: a crucial criterion for horse welfare?. Animal: an international journal of animal bioscience.

[24] Visser EK (2001). Quantifying aspects of young horses’ temperament consistency of behavioural variables. Applied Animal Behaviour Science.

[25] Nicol CJ (2005). The effects of diet and weaning method on the behaviour of young horses. Applied Animal Behaviour Science.

[26] Freire R, Clegg HA, Buckley P, Friend MA, McGreevy PD (2009). The effects of two different amounts of dietary grain on the digestibility of the diet and behaviour of intensively managed horses. Applied Animal Behaviour Science.

[27] Christensen JW, Keeling LJ, Nielsen BL (2005). Responses of horses to novel visual, olfactory and auditory stimuli. Applied Animal Behaviour Science.

[28] Daly K (2012). Alterations in microbiota and fermentation products in equine large intestine in response to dietary variation and intestinal disease. British Journal of Nutrition.

[29] Harlow BE, Lawrence LM, Hayes SH, Crum A, Flythe MD (2016). Effect of Dietary Starch Source and Concentration on Equine Fecal Microbiota. PloS one.

[30] Milinovich GJ (2008). Microbial ecology of the equine hindgut during oligofructose-induced laminitis. The ISME journal.

[31] van den Berg M, Hoskin SO, Rogers CW, Grinberg A (2013). Fecal pH and Microbial Populations in Thoroughbred Horses During Transition from Pasture to Concentrate Feeding. Journal of Equine Veterinary Science.

[32] Lyte M (2016). Resistant Starch Alters the Microbiota-Gut Brain Axis: Implications for Dietary Modulation of Behavior. PloS one.

[33] Hoban AE (2016). Behavioural and neurochemical consequences of chronic gut microbiota depletion during adulthood in the rat. Neuroscience.

[34] National Research Council, *Nutritional Requirements of Horses: Sixth Edition*. (The National Academies Press, 2007).

[35] Lansade L, Bouissou M-F (2008). Reactivity to humans: A temperament trait of horses which is stable across time and situations. Applied Animal Behaviour Science.

[36] Lansade L, Bouissou M-F, Erhard HW (2008). Fearfulness in horses: A temperament trait stable across time and situations. Applied Animal Behaviour Science.

[37] Wemelsfelder F, Hunter TEA, Mendl MT, Lawrence AB (2001). Assessing the ‘whole animal’: a free choice profiling approach. Animal Behaviour.

[38] Wemelsfelder F (2007). How animals communicate quality of life: the qualitative assessment of behaviour. Animal Welfare.

[39] Napolitano F (2008). The qualitative assessment of responsiveness to environmental challenge in horses and ponies. Applied Animal Behaviour Science.

[40] Wemelsfelder F, Hunter AE, Paul ES, Lawrence AB (2012). Assessing pig body language: Agreement and consistency between pig farmers, veterinarians, and animal activists. Journal of animal science.

[41] Fleming PA, Paisley CL, Barnes AL, Wemelsfelder F (2013). Application of Qualitative Behavioural Assessment to horses during an endurance ride. Applied Animal Behaviour Science.

[42] Minero M, Tosi MV, Canali E, Wemelsfelder F (2009). Quantitative and qualitative assessment of the response of foals to the presence of an unfamiliar human. Applied Animal Behaviour Science.

[43] Caporaso JG (2010). QIIME allows analysis of high-throughput community sequencing data. Nature Methods.

[44] R Core Team. R: A language and environment for statistical computing. R Foundation for Statistical Computing (Vienna, Austria, 2017).

[45] Zuur, A. F., Ieno, E. N., Walker, N. J., Saveliev, A. A. & Smith, G. N. *Mixed Effects Models and Extensions in Ecology with R*. (Springer Science + Business Media, 2009).

[46] nlme: Linear and Nonlinear Mixed Effects Models (R package version 3.1–131, 2017).

[47] Bates D, Maechler M, Bolker B, Walker S (2015). Fitting Linear Mixed-Effects Models Using lme4. Journal of Statistical Software.

[48] vegan: Community Ecology Package version 2.4–5 (2017).

[49] Vavrek MJ (2011). fossil: palaeoecological and palaeogeographical analysis tools. Palaeontologia Electronica.

[50] mvabund: Statistical Methods for Analysing Multivariate Abundance Data (R package version 3.12.3, 2017).

[51] Warton DI, Wright ST, Wang Y (2012). Distance-based multivariate analyses confound location and dispersion effects. Methods in Ecology and Evolution.

[52] Wang Y, Naumann U, Wright ST, Warton D (2012). I. mvabund- anRpackage for model-based analysis of multivariate abundance data.. Methods in Ecology and Evolution.

[53] Xu Q, Liang Y (2001). Monte Carlo cross validation. Chemometrics and Intelligent Laboratory Systems.

